# Assessing the priority of human rights and mental health: the PHRAME approach

**DOI:** 10.1192/bjo.2023.41

**Published:** 2023-03-27

**Authors:** Petra C. Gronholm, Neeraj Gill, Grace Carter, Danielle Watson, Hanfried Helmchen, Graham Thornicroft, Norman Sartorius

**Affiliations:** Centre for Global Mental Health, Health Service and Population Research Department, Institute of Psychiatry, Psychology and Neuroscience, King's College London, UK; and Centre for Implementation Science, Health Service and Population Research Department, Institute of Psychiatry, Psychology and Neuroscience, King's College London, UK; School of Medicine and Dentistry, Griffith University, Australia; Mental Health Policy Unit, Health Research Institute, University of Canberra, Australia; and Mental Health and Specialist Services, Gold Coast Health, Australia; School of Law, Faculty of Social Sciences, University of Nottingham, UK; and Institute of Mental Health, University of Nottingham, UK; Clinic for Psychiatry and Psychotherapy, Department of Psychiatry and Psychotherapy, Charité – University Medicine Berlin, Germany; Association of the Improvement of Mental Health Programs, Switzerland

**Keywords:** Human rights, psychiatry and law, patients, stigma and discrimination, expert consultation

## Abstract

**Background:**

Protecting all human rights of people with mental health conditions is globally important. However, to facilitate practical implementation of rights, it is often necessary to decide which of these rights should be given priority, especially when they conflict with each other.

**Aims:**

The aim of the Priorities of Human Rights and Mental Health (PHRAME) project is to develop a replicable approach to establish a proposed set of high-priority human rights of people with mental health conditions, to facilitate practical decision-making and implementation of such rights.

**Method:**

A two-stage Delphi-style study with stakeholders was conducted to generate a list of key rights of people with mental health conditions, and rank priorities among these rights in terms of feasibility, urgency and overall importance.

**Results:**

The stakeholders in this study consistently ranked three rights as top priorities: (a) the right to freedom from torture, cruel inhuman treatment and punishment; (b) the right to health and access to services/treatment; and (c) the right to protection and safety in emergency situations.

**Conclusions:**

Insights from PHRAME can support decision-making about the priority to be given to human rights, to guide practical action. This approach can also be used to assess how human rights are prioritised in different settings and by different stakeholders. This study identifies the clear need for a central voice for people with lived experience in research and implementation of decisions about the priority of human rights, ensuring that action respects the opinion of people whose rights are directly affected.

The current human rights framework was created by the United Nations through the Universal Declaration of Human Rights in 1948.^1^ This declaration is considered to be a foundational text in the history of human and civil rights, and this milestone document was the first to set out the basic human rights and fundamental freedoms that should be universally protected. In 1966, the International Covenant on Civil and Political Rights^2^ and the International Covenant on Economic, Social and Cultural Rights^3^ made it obligatory for signatory countries to protect and promote those human rights. However, there was no specific mention of people with disabilities in these documents. The rights of persons with mental illness received international acknowledgement through the Principles for the Protection of Persons with Mental Illness and the Improvement of Mental Health Care, also called the Mental Illness Principles, adopted by the United Nations General Assembly in December of 1991.^4^ However, the Mental Illness Principles did not have legally binding status in terms of international law.^5^ The United Nations Convention on the Rights of Persons with Disabilities (CRPD)^6^ is the first international treaty to set out human rights specifically for people with mental disabilities in international law. The CRPD incorporates contemporary human rights discourse, which includes economic, social and cultural rights, as well as civil and political rights.

In practice, it is often difficult to know how to prioritise and implement such rights; for example, because specific human rights are sometimes in tension or contradiction with others. Staff involved in mental healthcare and treatment often face dilemmas in knowing how to prioritise human rights when they cannot be implemented simultaneously. This includes decisions on what should be done if different sets of rights are in conflict with others, and considering to what extent the views of courts, mental health professionals and people with lived experience of mental health conditions are taken into account when resolving these conflicts. The Vienna Declaration and Programme of Action highlighted that all human rights are interrelated, interdependent and indivisible.^7^ Given the indivisibility and universality of human rights, there are different opinions regarding whether it is appropriate to set priorities among human rights. Some have argued against setting principled priorities among human rights, advocating for a focus on advancing full realisation of all human rights for all human beings.^8^ In doing so, Philips cautions against priority setting too quickly or taking a narrow view of human rights.^8^ Others, while accepting the substantive importance of all human rights, have advocated for a hierarchy at implementation level. Thereby, there can be progressive realisation of all human rights based on explicated shared priorities in the implementation process.^9^

## Aim and objectives

The Priorities of Human Rights and Mental Health (PHRAME) project sets out to compile a set of statements describing human rights of persons with mental health conditions, and to develop an approach to decision-making regarding human rights priorities of people with mental health conditions. This is envisioned to help and guide practical action in relation to efforts to protect and promote human rights, so that progress can be made to uphold human rights in harmony with the wishes and preferences of those whose human rights are concerned, in all situations.

The specific objectives of this PHRAME project are to:

- compile a comprehensive set of human rights statements of persons with mental health conditions;
- identify actionable, clear, tangible and measurable priorities among these rights – ranked in terms of feasibility, urgency and overall importance – explored across and between different stakeholder groups;
- develop a replicable approach for priority setting that can be used not only in this project, but also in other work examining priorities regarding human rights, so as to facilitate process of identifying actions to take and developing indicators that will help to assess the current state in relation to upholding human rights of people with mental health conditions.

## Method

This project uses a Delphi-style study method^10,11^ involving a two-stage process gathering data and feedback from experts. Specifically, we sought to identify, consolidate, score and rank priorities regarding human rights of people with mental health conditions.

The authors assert that all procedures contributing to this work comply with the ethical standards of the relevant national and institutional committees on human experimentation and with the Helsinki Declaration of 1975, as revised in 2008. All procedures involving human participants were approved by the Psychiatry, Nursing and Midwifery Research Ethics Subcommittee at King's College London (approval number HR-17/18-5521).

### Stage 1: generate synthesised list of key rights of people with mental health conditions

First, an exploratory literature review was conducted (by P.C.G., N.S. and G.T.) to identify materials that reflected human rights as specified in international law, including legislation referring to persons with mental health conditions and psychosocial disabilities, and human rights legislation. Inclusion was restricted to international laws published since 1948 (the publication of the Universal Declaration of Human Rights^1^).

Second, an expert panel was formed from globally leading figures in the fields of human rights, mental health law, public health, psychiatry, medical ethics, disability support advocacy and representation of people with lived experience of mental health conditions. This group (*N* = 21) was involved in the first step of expert consultations, where the literature review materials were reviewed and suggestions were provided for further relevant content.

Third, to generate a list of key rights, the list of 28 potentially relevant documents sourced through the exploratory searchers and expert recommendations was reviewed by colleagues with expertise in law and mental health (G.C. and D.W.). Twenty-three documents were identified for inclusion (see Supplementary File 1 available at https://doi.org/10.1192/bjo.2023.41 for further detail). This list of provisions is, we believe, a comprehensive list of rights accorded of persons with mental health conditions, but it is not exhaustive of all binding and non-binding documents that could be reasonably related to persons with mental health conditions and psychosocial disabilities. The relevant content regarding human rights of people with mental health conditions as expressed in each document was synthesised by G.C. and D.W., and formulated into one-sentence summaries. These summaries were reviewed by P.C.G., and grouped within similar thematic domains. Based on this synthesis, statements capturing these rights were generated, and these were reviewed by G.C. and D.W. Through subsequent iterative group discussions between G.C., D.W., N.G. and P.C.G., a final synthesised set of 21 statements reflecting key human rights of persons with mental health conditions was agreed.

### Stage 2: scoring and identifying priorities within the list of key rights

Experts were contacted to score key rights via an online survey. A snowballing approach was used: the survey link was sent to those who were contacted in the first stage of consultation, who were encouraged to disseminate the link further to their contacts. The link took prospective participants to a study information page, emphasising that participation in the individual and fully anonymous survey was voluntary, and that informed consent to participate was provided by clicking ‘next’ to proceed to the survey and clicking ‘submit’ once completed.

The survey outlined the statements of human rights of persons with mental health conditions generated in the previous stage of the project. Respondents were asked to indicate to which extent they agreed that upholding the specific rights was feasible, urgent and overall important, using a five-point Likert scale (1, disagree strongly; 2, disagree; 3, neutral (neither agree nor disagree); 4, agree; 5, agree strongly). See Table 1 for further detail on these criteria.

**Table 1:** The criteria used for scoring the statements on human rights of persons with mental illness

Feasibility	Ranking based on feasibility involves assessing the implementability/actionability of a given right regarding human rights among people with mental illness. In other words, is the ensuring that this right is upheld within the remit of a government or jurisdiction, could a single ministry or government action this? Is upholding this right something a government would see as achievable within a medium-term 3–5 year time span/a single electoral cycle? Could upholding this right be linked to existing legal frameworks (e.g. binding treaties or customary law) or international resolutions (e.g. United Nations Sustainable Development Goals) to facilitate translating the position into practice?
Urgency	Ranking based on urgency involves assessing which specific right regarding human rights among people with mental illness, among multiple other rights, should be attended to first. In other words, for which smaller number of rights should it be argued that governments should act on these sooner rather than later? For example, are there aspects of human rights that other rights depend on, or specific aspects that facilitate the implementation of subsequent rights? Which elements of human rights need to be attended to first in a staged process of implementation? Are there aspects within human rights statements, or violations of these, that are so fundamentally important that they need to be attended to immediately?
Overall importance	Ranking based on this criterion would combine elements of feasibility and urgency, as described above, and would involve an additional judgement of overall significance. In other words, the overall significance could depend on, for example, improvements in quality of life, or reflect priorities and preferences expressed by people with mental illness/disabilities, their families or organisations acting on their behalf.

Data were also collected on respondents’ gender, age, stakeholder affiliation, years of work experience/activity in their area of expertise, region of work (categorised as per World Bank regions^12^) and whether the respondent had any experience of caring for someone with mental health conditions. An open-ended field for feedback was provided for respondents to elaborate on their scoring rationale and provide other comments.

Mean scores were computed for the extent to which respondents agreed that each human rights statement was feasible, urgent and overall important. Priorities among these scores were identified by ranking the statements in terms of agreement. These scores reflecting to what extent stakeholders agreed that key human rights for people with mental health conditions were feasible, urgent and overall important were examined across the total sample, and between stakeholder subgroups (people with lived experience of mental health conditions, academic/researcher, clinician, other). To further examine the usefulness of this methodology to identify human rights priorities, a *post hoc* exploration was added to examine differences in these scores between participants working in different world regions (high income, other).

To explore differences between these scores, the Friedman test was used to compare rank distributions between related scores, and the Kruskal–Wallis test was used for comparisons between independent groups (tests chosen as data were not normally distributed). Where statistically significant differences in scores were identified when comparing three or more scores, the Wilcoxon signed-rank test (for related scores) and Dunn's pairwise test (for independent scores) were used to examine the differences, applying the Bonferroni correction to adjust for multiple testing. No further corrections for multiple comparisons were performed, given the exploratory, non-hypothesis testing nature of the analyses.^13,14^ Analyses were performed in Stata for Windows/MP version 16.0 and IBM SPSS for Windows version 25.

Open-ended feedback from respondents was reviewed, thematically synthesised and reported in a narrative format.

## Results

### Participant characteristics

A total of 71 experts were involved in scoring these 21 statements on human rights based on feasibility, urgency and overall importance. The sample had equal representation of men and women (48% *v*. 52%). A total of 47% of participants were aged 45–64 years, 29% were aged 25–44 years and 24% were aged 65 years or older. Participants had on average 22.8 years (s.d. 13.5) of work experience in their field (range 0–50 years). Most (60%) worked primarily in a high-income region. In terms of stakeholder affiliation, the three largest groups were academic/researcher (37%), person with lived experience of a mental health condition (24%) and clinician (23%). The remaining affiliations were combined in an ‘other’ category (advocates and family members 11%, policy makers 3%, ‘other’ 1%). The vast majority (90%) of respondents reported having experience of providing care for someone with a mental health condition. For most (63%), this was in a professional as opposed to personal capacity.

### Examining scores across whole sample

Table 2 displays the scores for the feasibility, urgency and overall importance for each of the 21 human rights statements, across the whole sample.

**Table 2:** Scores for the key human rights statements in terms of agreement with their feasibility, urgency and overall importance

Key human rights statements	Feasibility		Urgency		Overall importance		
	Mean	s.d.	Mean	s.d.	Mean	s.d.	*P-*value
1. Right to equality in all aspects of the law	4.00	1.03	4.27	0.81	4.43	0.78	**^a^
2. Right to freedom from discrimination in accessing rights on the grounds of disability	4.13	0.87	4.47	0.65	4.56	0.63	**^b^
3. Right to freedom from exploitation, violence and abuse	4.09	1.18	**4**.**77**	**0**.**57**	**4**.**84**	**0**.**44**	**^c^
4. Right to freedom from torture, cruel and inhuman treatment and punishment which would detrimentally impact mental well-being	**4**.**36**	**1**.**05**	**4**.**83**	**0**.**51**	**4**.**87**	**0**.**42**	**^c^
5. Right to equal recognition before the law (including equal legal capacity to hold and exercise rights and have decisions legally enforced)	3.81	1.11	4.14	0.90	4.27	0.95	**^c^
6. Right to effective access to justice, including accommodations to participate in justice and legal proceedings	4.10	1.01	4.35	0.72	4.44	0.69	*^d^
7. Right to health, including access to health services/appropriate treatment	**4**.**36**	**0**.**87**	**4**.**69**	**0**.**56**	**4**.**74**	**0**.**56**	**^c^
8. Right to consent to treatment	3.84	1.22	4.14	1.05	4.27	1.01	**^c^
9. Right to challenge potential rights violation before a judicial body or committee	4.16	1.03	4.35	0.78	4.49	0.70	*^a^
10. Right to work and workplace equality	3.88	1.02	4.27	0.69	4.48	0.64	**^b^
11. Right to provision of services and programmes that enable the attainment and maintenance of independence, capability, inclusion and participation in all aspects of life	3.86	1.20	4.39	0.77	4.59	0.75	**^b^
12. Right to education	4.17	0.94	4.39	0.77	4.49	0.66	*^d^
13. Right to adequate living standards	4.01	1.14	**4**.**54**	**0**.**66**	**4**.**64**	**0**.**59**	**^c^
14. Right to social inclusion and participation in community life (including right to (re)habilitation)	4.06	1.13	4.49	0.70	4.63	0.62	**^b^
15. Right to measures which facilitate independent living	3.80	1.11	4.28	0.73	4.39	0.75	**^c^
16. Right to personal physical mobility	3.94	0.98	4.25	0.75	4.43	0.63	**^b^
17. Right to participation in cultural life	3.93	1.08	4.04	0.94	4.26	0.87	**^a^
18. Right to participation in political life	3.88	1.08	4.07	0.85	4.24	0.81	**^d^
19. Right to protection and safety in emergency situations	**4**.**29**	**0**.**90**	**4**.**61**	**0**.**67**	**4**.**74**	**0**.**53**	**^b^
20. Right to freedom of expression, and access to information	**4**.**23**	**0**.**91**	4.37	0.75	4.51	0.68	**^a^
21. Right to consent to participate in research	**4**.**39**	**0**.**87**	4.35	0.81	4.47	0.72	

Bolding indicates the top five items with strongest agreement. Footnote citations show patterns of differences in scores.

a. Scores for urgency and feasibility are statistically comparable, but statistically significantly lower than the score for overall importance.

b. All three scores are statistically significantly different from each other; feasibility is ranked lowest and overall importance highest.

c. The feasibility score is statistically significantly lower than the scores for either urgency or overall importance; the latter two are statistically comparable.

d. Scores for feasibility are statistically significantly lower than the scores for overall importance; no difference between scores for urgency and either feasibility overall importance.

*P ≤ 0.05; **P ≤ 0.01.

Focusing on the top five statements in terms of priority, three rights were ranked in this top group across all three scoring domains (feasibility, urgency and overall importance): ‘right to freedom from torture, cruel inhuman treatment and punishment’; ‘right to health and access to services/treatment’ and ‘right to protection and safety in emergency situations’.

When exploring differences in rank distribution across the three scoring domains, the scores for feasibility were lowest, and scores for overall importance were highest.

Regarding feasibility, the pattern of statistically significant differences in scores indicated that for each right statement where there was a difference in scores, the score for agreement with the feasibility of a given right was significantly lower than the scores for agreeing with overall importance and urgency, or scores for overall importance only.

Regarding urgency, agreement with this was generally lower than agreement with overall importance, and either the same as or higher than agreement with feasibility.

Regarding overall importance, agreement with this was either higher than agreement with both feasibility and urgency of a given right, or higher than agreement with feasibility only.

### Examining scores between stakeholder groups

Tables 3–5 display scores indicating agreement on the feasibility, urgency and overall importance for each human rights statement per participant stakeholder group (people with lived experience of mental health conditions, academic/researcher, clinician, other).

**Table 3:** Key human rights of people with mental illness, as assessed in terms of feasibility, arranged per key stakeholder group

Key human rights statements	People with lived experience of mental health conditions (*n* = 17)		Academic/ researcher (*n* = 26)		Clinician (*n* = 16)		Other (*n* = 11)		
	Mean	s.d.	Mean	s.d.	Mean	s.d.	Mean	s.d.	*P*-value
1. Right to equality in all aspects of the law	4.38	0.72	3.56	1.12	4.38	0.96	4.09	0.83	^a^
2. Right to freedom from discrimination in accessing rights on the grounds of disability	4.41	0.87	**3**.**92**	**0**.**81**	4.31	0.87	4.09	0.70	
3. Right to freedom from exploitation, violence and abuse	4.35	1.17	3.52	1.23	**4**.**63**	**0**.**72**	**4**.**45**	**0**.**69**	**^b^
4. Right to freedom from torture, cruel and inhuman treatment and punishment which would detrimentally impact mental well-being	4.47	0.94	**3**.**92**	**1**.**19**	**4**.**69**	**1**.**01**	**4**.**73**	**0**.**65**	*^b^
5. Right to equal recognition before the law (including equal legal capacity to hold and exercise rights and have decisions legally enforced)	4.18	0.95	3.44	1.16	4.13	1.02	3.82	1.08	
6. Right to effective access to justice, including accommodations to participate in justice and legal proceedings	4.47	0.62	**3**.**88**	**1**.**09**	4.06	1.06	4.36	0.67	
7. Right to health, including access to health services/appropriate treatment	**4**.**56**	**0**.**63**	3.88	0.97	**4**.**88**	**0**.**34**	**4**.**64**	**0**.**67**	**^b^^,^^c^
8. Right to consent to treatment	4.41	0.71	3.48	1.39	4.06	1.06	3.45	1.44	
9. Right to challenge potential rights violation before a judicial body or committee	**4**.**71**	**0**.**47**	3.88	1.09	4.25	1.06	4.00	1.10	
10. Right to work and workplace equality	**4**.**53**	**0**.**62**	3.48	1.16	3.75	0.86	4.20	0.79	**^d^
11. Right to provision of services and programmes that enable the attainment and maintenance of independence, capability, inclusion and participation in all aspects of life	4.41	0.80	3.32	1.28	3.81	1.28	**4**.**45**	**0**.**69**	*^d^
12. Right to education	4.44	0.96	3.76	0.88	**4**.**56**	**0**.**81**	4.36	0.67	**^b^^,^^d^
13. Right to adequate living standards	4.41	1.00	3.68	1.11	4.19	1.22	4.20	0.79	
14. Right to social inclusion and participation in community life (including right to (re)habilitation)	4.35	0.86	3.52	1.29	4.38	1.09	4.36	0.81	^a^
15. Right to measures which facilitate independent living	4.24	0.97	3.76	1.05	3.47	1.25	3.82	1.08	
16. Right to personal physical mobility	4.47	0.87	3.68	0.99	3.80	1.08	4.09	0.54	*^d^
17. Right to participation in cultural life	4.24	0.90	3.58	1.21	3.93	1.16	4.18	0.87	
18. Right to participation in political life	4.50	0.73	3.76	1.09	3.63	1.26	3.82	0.87	
19. Right to protection and safety in emergency situations	**4**.**65**	**0**.**61**	**3**.**96**	**1**.**10**	4.44	0.63	4.45	0.69	
20. Right to freedom of expression, and access to information	4.47	0.80	3.84	0.90	4.44	0.89	**4**.**70**	**0**.**48**	**^c^
21. Right to consent to participate in research	**4**.**53**	**0**.**72**	**4**.**16**	**0**.**99**	**4**.**63**	**0**.**89**	4.36	0.81	

Bolding indicates the top five items with strongest agreement. Footnote citations show patterns of differences in scores.

a. No differences remained when adjusting for multiple testing.

b. Academic/researcher score significantly lower than clinician.

c. Academic/researcher score significantly lower than ‘other’.

d. Academic/researcher score significantly lower than persons with lived experience.

*P ≤ 0.05; **P ≤ 0.01.

**Table 4:** Key human rights of people with mental illness, as assessed in terms of urgency, arranged per key stakeholder group

Key human rights statements	People with lived experience of mental health conditions (*n* = 17)		Academic/ researcher (*n* = 26)		Clinician (*n* = 16)		Other (*n* = 11)		
	Mean	s.d.	Mean	s.d.	Mean	s.d.	Mean	s.d.	*P*-value
1. Right to equality in all aspects of the law	**4**.**79**	**0**.**43**	4.00	0.91	4.31	0.48	4.18	1.08	*^a^
2. Right to freedom from discrimination in accessing rights on the grounds of disability	4.53	0.87	4.36	0.57	**4**.**56**	**0**.**51**	4.55	0.69	
3. Right to freedom from exploitation, violence and abuse	**4**.**76**	**0**.**75**	**4**.**72**	**0**.**54**	**5**.**00**	**0**.**00**	**4**.**64**	**0**.**67**	
4. Right to freedom from torture, cruel and inhuman treatment and punishment which would detrimentally impact mental well-being	**4**.**82**	**0**.**53**	**4**.**73**	**0**.**67**	**4**.**94**	**0**.**25**	**4**.**91**	**0**.**30**	
5. Right to equal recognition before the law (including equal legal capacity to hold and exercise rights and have decisions legally enforced)	4.41	0.80	3.96	1.04	4.25	0.58	4.18	0.87	
6. Right to effective access to justice, including accommodations to participate in justice and legal proceedings	**4**.**76**	**0**.**44**	4.21	0.66	4.25	0.77	4.36	0.67	*^a^
7. Right to health, including access to health services/appropriate treatment	4.69	0.60	**4**.**58**	**0**.**50**	**4**.**75**	**0**.**58**	**4**.**80**	**0**.**63**	
8. Right to consent to treatment	4.53	0.72	4.04	1.12	4.06	1.00	3.91	1.38	
9. Right to challenge potential rights violation before a judicial body or committee	4.76	0.44	4.17	0.82	4.44	0.63	4.18	0.87	
10. Right to work and workplace equality	4.59	0.51	4.08	0.78	4.13	0.62	4.44	0.73	
11. Right to provision of services and programmes that enable the attainment and maintenance of independence, capability, inclusion and participation in all aspects of life	4.53	0.62	4.25	0.94	4.25	0.58	**4**.**64**	**0**.**81**	
12. Right to education	4.65	0.61	4.17	0.82	4.44	0.81	4.45	0.82	
13. Right to adequate living standards	**4**.**82**	**0**.**39**	**4**.**50**	**0**.**66**	4.38	0.72	4.60	0.70	
14. Right to social inclusion and participation in community life (including right to (re)habilitation)	4.65	0.61	4.38	0.71	**4**.**50**	**0**.**73**	4.55	0.82	
15. Right to measures which facilitate independent living	4.53	0.62	4.29	0.62	4.13	0.74	4.09	1.04	
16. Right to personal physical mobility	4.65	0.61	4.09	0.73	4.13	0.92	4.18	0.60	
17. Right to participation in cultural life	4.25	0.93	3.83	1.05	4.13	0.83	4.09	0.94	
18. Right to participation in political life	4.63	0.72	3.83	0.87	4.00	0.82	3.91	0.83	*^a^
19. Right to protection and safety in emergency situations	4.59	0.71	**4**.**71**	**0**.**55**	4.50	0.82	**4**.**64**	**0**.**67**	
20. Right to freedom of expression, and access to information	4.71	0.47	4.25	0.68	4.31	0.87	4.40	0.70	
21. Right to consent to participate in research	4.41	0.80	4.29	0.75	4.47	0.74	4.45	0.82	

Bolding indicates the top five items with strongest agreement. Footnote citations show patterns of differences in scores.

a. Academic/researcher score significantly lower than people with lived experience of mental health conditions.

*P ≤ 0.05.

**Table 5:** Key human rights of people with mental illness, as assessed in terms of overall importance, arranged per key stakeholder group

Key human rights statements	People with lived experience of mental health conditions (*n* = 17)		Academic/ researcher (*n* = 26)		Clinician (*n* = 16)		Other (*n* = 11)		
	Mean	s.d.	Mean	s.d.	Mean	s.d.	Mean	s.d.	*P-*value
1. Right to equality in all aspects of the law	**4**.**88**	**0**.**34**	4.15	0.92	4.63	0.50	4.30	0.82	*^a^
2. Right to freedom from discrimination in accessing rights on the grounds of disability	4.59	0.87	4.54	0.51	4.63	0.50	4.55	0.69	
3. Right to freedom from exploitation, violence and abuse	**4**.**94**	**0**.**25**	**4**.**81**	**0**.**40**	**5**.**00**	**0**.**00**	**4**.**73**	**0**.**65**	
4. Right to freedom from torture, cruel and inhuman treatment and punishment which would detrimentally impact mental well-being	**4**.**88**	**0**.**49**	**4**.**75**	**0**.**53**	**5**.**00**	**0**.**00**	**5**.**00**	**0**.**00**	
5. Right to equal recognition before the law (including equal legal capacity to hold and exercise rights and have decisions legally enforced)	4.59	0.87	4.12	1.09	4.38	0.62	4.18	0.87	
6. Right to effective access to justice, including accommodations to participate in justice and legal proceedings	4.65	0.61	4.36	0.64	4.63	0.50	4.27	0.79	
7. Right to health, including access to health services/appropriate treatment	4.69	0.70	**4**.**64**	**0**.**57**	**4**.**94**	**0**.**25**	**4**.**80**	**0**.**63**	
8. Right to consent to treatment	4.65	0.61	4.16	1.11	4.25	0.86	4.00	1.41	
9. Right to challenge potential rights violation before a judicial body or committee	**4**.**82**	**0**.**39**	4.36	0.70	4.56	0.63	4.36	0.67	
10. Right to work and workplace equality	4.80	0.41	4.20	0.71	4.56	0.51	4.60	0.70	*^a^
11. Right to provision of services and programmes that enable the attainment and maintenance of independence, capability, inclusion and participation in all aspects of life	4.71	0.59	4.40	0.96	**4**.**75**	**0**.**45**	**4**.**64**	**0**.**81**	
12. Right to education	4.71	0.59	4.28	0.68	4.63	0.62	4.50	0.71	
13. Right to adequate living standards	4.71	0.47	**4**.**60**	**0**.**58**	4.69	0.60	**4**.**70**	**0**.**67**	
14. Right to social inclusion and participation in community life (including right to (re)habilitation)	4.71	0.59	4.60	0.58	4.63	0.62	4.64	0.81	
15. Right to measures which facilitate independent living	4.65	0.61	4.36	0.64	4.33	0.82	4.27	1.01	
16. Right to personal physical mobility	4.76	0.56	4.28	0.61	4.53	0.52	4.27	0.65	*^a^
17. Right to participation in cultural life	4.35	0.93	4.00	0.96	4.60	0.51	4.27	0.90	
18. Right to participation in political life	4.65	0.70	4.04	0.73	4.19	0.83	4.27	0.90	
19. Right to protection and safety in emergency situations	**4**.**88**	**0**.**49**	**4**.**72**	**0**.**54**	**4**.**75**	**0**.**45**	4.64	0.67	
20. Right to freedom of expression, and access to information	4.82	0.39	4.36	0.64	4.63	0.62	4.40	0.70	
21. Right to consent to participate in research	4.41	0.80	4.40	0.65	4.75	0.45	4.55	0.69	

Bolding indicates the top five items with strongest agreement. Footnote citations show patterns of differences in scores.

a. Academic/researcher score significantly lower than persons with lived experience.

*P ≤ 0.05.

The top five priority statements indicated limited consistency in agreement regarding feasibility across the stakeholder groups. No human right was a top five priority across all groups, but ‘the right to health and access to services/treatment’ was given high priority in feasibility by people with lived experience of mental health conditions, clinicians and others; ‘the right to freedom from torture, cruel inhuman treatment and punishment’ was prioritised in feasibility by academics/researchers, clinicians and others; and ‘the right to consent to participate in research’ was prioritised in feasibility by people with lived experience of mental health conditions, academics/researchers and clinicians.

In terms of urgency, the top five scores were more consistent across the stakeholder groups. The rights to ‘freedom from torture, cruel inhuman treatment and punishment’ and ‘freedom from exploitation, violence and abuse’ were prioritised by all groups. In addition, the ‘right to health and access to services/treatment’ was prioritised by academics/researchers, clinicians and the ‘other’ group. These rights were prioritised in the same way in relation to overall importance. Additionally, the right to ‘protection and safety in emergency situations’ was prioritised for overall importance by people with lived experience of mental health conditions, academics/researchers and clinicians.

When exploring differences in rank distribution across these data, where statistically significant differences were observed between the scores for different stakeholder groups, this always reflected lower scores of agreement on feasibility, urgency or overall importance by academics/researchers as compared with another stakeholder group. On ten occasions these differences were between scores of academics/researchers and people with lived experience of mental health conditions, followed by four differences with clinicians and two with the ‘other’ group.

Regarding agreement on feasibility, academics/researchers’ scores were lower than clinicians’ for rights to ‘freedom from exploitation, violence and abuse’; ‘freedom from torture, cruel inhuman treatment and punishment’; ‘health and access to services/treatment’ and ‘education’. Academics/researchers’ scores were also lower than people with lived experience of mental health conditions regarding agreement on feasibility for ‘rights to work and workplace equality’; provisions that enable ‘independence, capability, inclusion and participation’; ‘education’ and ‘personal physical mobility’. Finally, academics/researchers’ scores were lower than the ‘other’ group for rights to ‘health and access to services/treatment’ and ‘freedom of expression and access to information’.

Regarding agreement on urgency, academics/researchers scored lower than people with lived experience of mental health conditions for rights to ‘equality in all aspects of the law’, ‘access to and participation in justice’ and ‘participation in political life’. Regarding overall importance, academics/researchers’ scores were lower than people with lived experience of mental health conditions for rights to ‘equality in all aspects of the law’, ‘work and workplace equality’ and ‘personal physical mobility’.

### Examining scores between participants working in different world regions

As a *post hoc* exploration we examined scores for feasibility, urgency and overall importance for each human rights statement, arranged based on participants’ region of work (high income versus other). The results table is available as Supplementary File 2.

Priorities were broadly comparable across the different scoring domains and work regions. Rights to ‘freedom from torture, cruel inhuman treatment and punishment’; ‘health and access to services/treatment’ and ‘protection and safety in emergency situations’ were among the top five priorities in terms of feasibility, urgency and overall importance for participants working in from both high-income regions and other world regions. Furthermore, both groups rated the ‘right to consent to participate in research’ as a top five priority for feasibility, and the ‘right to freedom from exploitation, violence and abuse’ as a top five priority for urgency and overall importance.

Where statistically significant differences were observed between the scores from different work region groups, this reflected higher scores of agreement on urgency or overall importance by participants working in high-income regions, as compared with ‘other’ regions. There were no differences between these groups for scores on feasibility.

### Narrative feedback

Narrative feedback was provided by 24 respondents. This group was broadly balanced by gender (54% female *v*. 46% male), and about half were aged 45–64 years (54% compared with 14% aged 25–44 years and 33% aged ≥65 years). The main reported stakeholder affiliations were academic/researcher (39%), advocate (26%) and person with lived experience of mental health condition (22%). Respondents had an average of 24.4 years of work experience in their field, with just over half (58%) working primarily in a high-income region. Nearly all (91%) had experience of providing care for someone with a mental health condition in a personal or professional capacity.

Their comments emphasised that priorities are likely to differ across settings and contexts, often requiring a local approach. The feedback also covered that care should be taken so decisions are not driven by feasibility and costs, but informed by what issues are meaningful. Engagement with key local stakeholders, and in particular with people with lived experience of mental health conditions and their families, is critically important in decision-making regarding priorities. Surveys were considered insufficient to capture an understanding of relevant priorities, and additional qualitative research was recommended. Respondents reflected on potential conflicts between universal legal capacity to hold rights versus capacity to consent under periods of significant acute illness, and also commented on conflicts between legal rights and practical reality (e.g. access to care if there are limited services available).

## Discussion

This project aimed to compile a set of statements describing human rights of persons with mental health conditions, and to establish an approach to assess the priority given to these rights by an expert stakeholder group. A reliable approach for such priority setting, according to set criteria, can be used by to identify actions and indicators to use in relation to protecting and promoting the human rights of people with mental health conditions.

In this project, we consolidated detailed international law documentation into a comprehensible core set of statements, reflecting the range of human rights of people with mental health conditions. There was general consensus among expert stakeholders involved in this study about the ways to interpret international legislation and statements pertaining to the human rights of persons with mental health conditions, from which the list of 21 human rights statement was generated.

In exploring priorities across the study sample, these three rights were consistently ranked as top priorities: (a) the right to freedom from torture, cruel inhuman treatment and punishment; (b) the right to health and access to services/treatment and (c) the right to protection and safety in emergency situations. This consistency indicates that, at least among this sample, these are critically important targets of action. Such insights are valuable to guide focus of action. Unlike debates in human rights literature about the importance of the so-called negative rights (restricting persons; civil and political rights) versus positive rights (providing persons with something; economic, social and cultural rights),^15^ our study found a mix of positive and negative rights as top priorities, affirming the importance of both.

The right to health and access to services/treatment has been highlighted by many scholars in the field of mental health. They argue that without a fundamental commitment of governments to satisfy basic health needs, including mental health, other rights are less meaningful and attainable.^16^ Economic, social and cultural rights are also often neglected in legislation and public policy because governments worry about legal implications if those rights are legislated, but not implemented because of resource constraints.^17^ They are perceived to be too costly, only reflecting interests of politically powerless and voiceless groups of people.^16^ However, a neglect of positive rights, which are the social determinants of mental health, leads to greater prevalence and poorer outcomes of mental health conditions.^18,19^ Therefore, legal framework and policy priorities must focus on economic, social and cultural rights and provision of accessible and high-quality, rights-based mental health and support services.^17,20^ This aligns with the high priority for the right to health and access to services/treatment identified in our study.

Scores for rights’ urgency and overall importance were often comparable, whereas ratings for feasibility differed. There was also often lower agreement with feasibility compared with urgency or overall importance. This might reflect an attitudinal bias of respondents accepting the status quo – that rights which were considered urgent/important were not considered feasible. It may also reflect a realisation of resource constraints and lack of priority given to those rights in public policy. This then leads to widespread violation of human rights of people with psychosocial disabilities, as has been documented previously.^21^ These disparities in scores indicate the importance of advocacy and struggle by all of the stakeholders to demand policy-making and resource-allocation targetted on improving feasibility to promote and protect urgent/important human rights and realisation of full citizenship.^22^ In this context, it has been argued that social citizenship rights include access to social and health services.^23^

Exploring feasibility, urgency and overall importance in the light of the opinions of people with mental health conditions specifically gives meaning to work that is conducted. For example, if in a given context the realisation of a particular right is considered highly feasible, but of lesser priority for overall importance and urgency, it might be more useful to change the focus of work to another human right that is considered both feasible and important by people with mental health conditions. Such strategic planning to ensure work conducted is as impactful as possible is critically important, particularly as work to support human rights is often conducted in a context of limited resources and facing other practical constraints, which makes it necessary to carefully select the focus for the work in a specific setting.

In terms of findings relating to different stakeholder groups, we observed no clear consensus in priorities between the groups we considered. These differences in scores from different stakeholder groups reflect the critical importance of ensuring that any work conducted in the field of human rights of people with mental health conditions is steered by diverse, interdisciplinary, multisectoral teams that include representatives of people with experience of mental health conditions, as well as other stakeholders.

Academics/researchers ranked many rights differently from people with lived experience. This highlights the importance of the voice of those with lived experience in research. In particular, the right to ‘equality in all aspects of the law’ was ranked significantly higher in urgency and overall importance by people with lived experience compared with academics/researchers. The right to equal recognition before the law, as highlighted by Article 12 of the United Nations CRPD, has significant implications on a range of legal frameworks, including mental health laws, guardianship laws, unsoundness of mind defence, and civil and criminal trial procedures.^24^ It has been at the heart of debate regarding involuntary psychiatric treatment.^17^ The call for a shift from a ‘best interest paradigm’ to the ‘will and preference of the individual paradigm’ arises from this right.^25^

The finding that people with lived experience ranked the right to equality before the law significantly higher than academics/researchers is striking, and shows the level of prevailing disagreement in this regard. This highlights the importance of research focused on lived experience, and of making lived experience central to decision-making in policy-making and clinical practice. To achieve meaningful change, the priorities to promote and protect human rights must be driven by persons with lived experience of mental health conditions.

It is also notable that the views of persons with lived experience aligned more with clinicians than academics/researchers. Qualitative insights could help explain why researchers’ views were often less optimistic with respect to feasibility, compared with other stakeholders.

Our *post hoc* exploration focused on respondents’ region of work. The consistency in rights prioritised between those working in high-income versus other regions indicated that comparable aspects of human rigths are observed globally. To understand diverse views in priorities, it seems necessary to consider these in terms of more detailed characteristics (e.g. stakeholder affiliation) than region of work.

### Strengths and limitations

This project set out to formulate a synthesised overview of human rights of people with mental health conditions, and an approach to explore priority setting of these rights. This priority setting approach helps to identify key domains on which to focus, to support decision-making and guide action in work protecting human rights. Our findings provide important insights into how human rights were prioritised by the different categories of participants of the expert group involved in the study (considering the scoring criteria of feasibility, urgency and overall importance), and a method to be used in in future work with other groups of respondents, exploring populations and priority domains that are meaningful to their work and context.

The main limitation was that for the stakeholder group analyses, we had to group a number of stakeholders into the ‘other’ category, as our sample size limited our ability to consider these groups separately. It would have been particularly useful to have had a large enough policy maker group to understand their priorities, given the key role policy makers hold in upholding and shaping legislation and related proceedings. Also, in our *post hoc* analyses on the impact of region of work, we had to group respondents from a diverse range of regions globally into a single ‘other’/non-high-income group. It is possible that this approach masked differences that might be present between people working in different low-to-middle income regions, and also has an effect on sample representativeness. Overall, the sampling approach in this study was not unbiased (expert invitations and snowballing). However, this is not considered an issue as this study did not seek a random representative sample and there is no intent to suggest the findings are generalisable (rather, they exemplify rankings within this specific stakeholder group). Another study limitation is that participant characteristic data were not collected during the first stage of expert contact, meaning that the sample could not be characterised. These details are, however, available to characterise the broader group involved in the second stage of the expert consultation, and this broader expert panel is based on the initial stakeholder group. This study did, however, provide an initial exploration of potential patterns in priorities of human rights as broadly considered across high-income versus other regions of activity. It also needs to be recognised that where differences in priority scores were observed, these were often small, albeit significant. When planning action it is often necessary to select one area of work and give it priority over another. The approach we established through the PHRAME project suggests how such choices can be made through a structured and strategic approach to establish a rank order of priorities of actions in the area of human rights, given the priorities identified in a given setting, context and/or group of key stakeholders.

In conclusion, there was consensus among different stakeholders in this sample about the array of human rights of people with mental health conditions contained in international legislation and statements. Three rights were consistently ranked in the top priority scores: (a) the right to freedom from torture, cruel inhuman treatment and punishment; (b) the right to health and access to services/treatment and (c) the right to protection and safety in emergency situations. These may be considered clear actionable priorities, which are most often urgent, very important and considered feasible.

Many other rights were considered urgent and important, but less feasible; for example, the rights to ‘adequate living standards’ and ‘freedom from exploitation, violence and abuse’. We recommend that policy action and resource allocation is directed to improve the feasibility of these rights and others that are considered urgent and important by stakeholders in a given setting and context.

When different sets of rights are in direct conflict, it is difficult to give priority to the implementation of a particular right over others. However, such decisions have to be made when action is required, and some guidance or consensus is therefore needed. This study proposes criteria that can help to make decisions and calls for the centrality of the opinion of people with lived experience in prioritising their human rights. Decisions about the priority of respecting human rights, in policy as well as practice, must respect the opinion of those whose rights are directly affected.

## Data Availability

The data that support the findings of this study are available on request from the corresponding author, P.C.G. Consent was not sought to share data publicly.

## Supplementary material

For supplementary material accompanying this paper visit http://doi.org/10.1192/bjo.2023.41. click here to view supplementary material
