# Supplementary material for: Assessing the priority of human rights and mental health: the PHRAME approach
Source: BJPsych Open. 2023 Mar 27;9(2):e56. doi: 10.1192/bjo.2023.41 (PMC10134285; doi:10.1192/bjo.2023.41)
Supplement: Supplementary file 1 [file bjosup.zip › S2056472423000418sup001.docx]

**Assessing the priority of human rights: the PHRAME approach**

Petra C Gronholm, Neeraj S Gill, Grace Carter, Danielle Watson, Hanfried Helmchen, Graham Thornicroft*, Norman Sartorius* (*Joint senior authors)

Corresponding author: Petra Gronholm, email: [petra.gronholm@kcl.ac.uk](mailto:petra.gronholm@kcl.ac.uk)

# Supplementary Material file 1: Documents meeting inclusion criteria

Binding documents with relevant provisions, and non-binding documents with relevant provisions or which provide authoritative interpretations for domestic law.

|  | Universal Declaration of Human Rights (1948, United Nations) |
| --- | --- |
|  | International Covenant on Economic, Social and Cultural Rights (1966, United Nations) |
|  | International Covenant on Civil and Political Rights (1966, United Nations) |
|  | Convention on the Rights of the Child (1989, United Nations) |
|  | Convention on the Rights of Persons with Disabilities (2006, United Nations.) |
|  | Optional Protocol to the Convention on the Rights of Persons with Disabilities (2006, United Nations) |
|  | General comment No. 1 Article 12: Equal recognition before the law (CRPD) (2014, Committee on the Rights of Persons with Disabilities) |
|  | Standard Minimum Rules for the Treatment of Prisoners (1955, United Nations) |
|  | Universal Declaration on the Human Genome and Human Rights (1997, UNESCO) |
|  | Declaration on the Rights of Disabled Persons (1975, United Nations) |
|  | General Comment No. 14: The Right to the Highest Attainable Standard of Health (Art. 12 of the Covenant) (2000, UN Committee on Economic, Social and Cultural Rights) |
|  | International Ethical Guidelines for Biomedical Research Involving Human Subjects (2002, World Health Organization) |
|  | Principles for the Protection of Persons with Mental Illness and the Improvement of Mental Health Care (1991, United Nations) |
|  | Guidelines for the promotion of human rights of persons with mental disorders (1996, World Health Organization) |
|  | Mental Health Care Law: Ten Basic Principles (1996, World Health Organization) |
|  | ICESCR Committee General comment No.5 Persons with Disabilities (9 December 1994, contained in Document E/1995/22) |
|  | General Assembly resolution 46/119 (1991) ‘Principles for the Protection of Persons with Mental Illness and the Improvement of Mental Health Care |
|  | Economic and Social Council resolution 1921 (LVIII) of 6 May 1975 on the prevention of disability and the rehabilitation of disabled persons |
|  | Economic and social council Declaration on Social Progress (GA resolution 2542 (XXIV) of 11 December 1969) |
|  | General Assembly resolution 2856 (XXVI) of 20 December 1971 ‘Declaration on the Rights of Mentally Retarded Persons’ |
|  | World Programme of action concerning disabled persons adopted by the GA in resolution 37/52 dated 3 December 1982 |
|  | International Labour Organizations Convention No. 159 (1983) concerning vocational rehabilitation and employment of persons with disabilities |
|  | Standard Rules on the Equalization of Opportunities for Persons with Disabilities, annexed to General Assembly resolution 48/96 of 20 December 1993 |
