# Supplementary material for: Assessing the priority of human rights and mental health: the PHRAME approach
Source: BJPsych Open. 2023 Mar 27;9(2):e56. doi: 10.1192/bjo.2023.41 (PMC10134285; doi:10.1192/bjo.2023.41)
Supplement: Supplementary file 1 [file bjosup.zip › S2056472423000418sup002.pdf]

Assessing the priority of human rights: the PHRAME approach

Petra C Gronholm, Neeraj S Gill, Grace Carter, Danielle Watson, Hanfried Helmchen, Graham Thornicroft*, Norman Sartorius* (*Joint senior authors)
Corresponding author: Petra Gronholm, email: petra.gronholm@kcl.ac.uk

Supplementary Material file 1: Documents meeting inclusion criteria

Binding documents with relevant provisions, and non-binding documents with relevant provisions or which provide authoritative interpretations for domestic law.

1.	Universal Declaration of Human Rights (1948, United Nations)
2.	International Covenant on Economic, Social and Cultural Rights (1966, United Nations)
3.	International Covenant on Civil and Political Rights (1966, United Nations)
4.	Convention on the Rights of the Child (1989, United Nations)
5.	Convention on the Rights of Persons with Disabilities (2006, United Nations.)
6.	Optional Protocol to the Convention on the Rights of Persons with Disabilities (2006, United Nations)
7.	General comment No. 1 Article 12: Equal recognition before the law (CRPD) (2014, Committee on the Rights of Persons with Disabilities)
8.	Standard Minimum Rules for the Treatment of Prisoners (1955, United Nations)
9.	Universal Declaration on the Human Genome and Human Rights (1997, UNESCO)
10.	Declaration on the Rights of Disabled Persons (1975, United Nations)
11.	General Comment No. 14: The Right to the Highest Attainable Standard of Health (Art. 12 of the Covenant) (2000, UN Committee on Economic, Social and Cultural Rights)
12.	International Ethical Guidelines for Biomedical Research Involving Human Subjects (2002, World Health Organization)
13.	Principles for the Protection of Persons with Mental Illness and the Improvement of Mental Health Care (1991, United Nations)
14.	Guidelines for the promotion of human rights of persons with mental disorders (1996, World Health Organization)
15.	Mental Health Care Law: Ten Basic Principles (1996, World Health Organization)
16.	ICESCR Committee General comment No.5 Persons with Disabilities (9 December 1994, contained in Document E/1995/22)
17.	General Assembly resolution 46/119 (1991) ‘Principles for the Protection of Persons with Mental Illness and the Improvement of Mental Health Care
18.	Economic and Social Council resolution 1921 (LVIII) of 6 May 1975 on the prevention of disability and the rehabilitation of disabled persons
19.	Economic and social council Declaration on Social Progress (GA resolution 2542 (XXIV) of 11 December 1969)
20.	General Assembly resolution 2856 (XXVI) of 20 December 1971 ‘Declaration on the Rights of Mentally Retarded Persons’
21.	World Programme of action concerning disabled persons adopted by the GA in resolution 37/52 dated 3 December 1982
22.	International Labour Organizations Convention No. 159 (1983) concerning vocational rehabilitation and employment of persons with disabilities
23.	Standard Rules on the Equalization of Opportunities for Persons with Disabilities, annexed to General Assembly resolution 48/96 of 20 December 1993
