# Supplementary material for: Assessing the priority of human rights and mental health: the PHRAME approach
Source: BJPsych Open. 2023 Mar 27;9(2):e56. doi: 10.1192/bjo.2023.41 (PMC10134285; doi:10.1192/bjo.2023.41)
Supplement: Supplementary file 1 [file bjosup.zip › S2056472423000418sup003.docx]

**Assessing the priority of human rights: the PHRAME approach**

Petra C Gronholm, Neeraj S Gill, Grace Carter, Danielle Watson, Hanfried Helmchen, Graham Thornicroft*, Norman Sartorius* (*Joint senior authors)

Corresponding author: Petra Gronholm, email: [petra.gronholm@kcl.ac.uk](mailto:petra.gronholm@kcl.ac.uk)

**Supplementary Material file 2: Post-hoc analysis results**

| Table: Key human rights of people with mental illness, as assessed in terms of feasibility, urgency, and overall importance, across the whole sample, arranged per participants’ region of work. ***Bold italics*** = top five scores with strongest agreement. *<0.05; **<0.01 | | | | | | | | | | | | | | | |
| --- | --- | --- | --- | --- | --- | --- | --- | --- | --- | --- | --- | --- | --- | --- | --- |
| Key human rights statements | FEASIBILITY | | | | | URGENCY | | | | | OVERALL IMPORTANCE | | | | |
|  | High income | | Other | |  | High income | | Other | |  | High income | | Other | |  |
|  | mean | SD | mean | SD | p | mean | SD | mean | SD | p | mean | SD | mean | SD | p |
| 1. Right to equality in all aspects of the law | 3.93 | 1.07 | 4.15 | 0.99 |  | 4.31 | 0.86 | 4.19 | 0.74 |  | 4.46 | 0.84 | 4.35 | 0.69 |  |
| 2. Right to freedom from discrimination in accessing rights on the grounds of disability | 4.13 | 0.82 | 4.19 | 0.88 |  | 4.58 | 0.59 | 4.37 | 0.56 |  | 4.66 | 0.53 | 4.48 | 0.58 |  |
| 3. Right to freedom from exploitation, violence, and abuse | 4.00 | 1.13 | 4.30 | 1.14 |  | ***4.78*** | ***0.53*** | ***4.85*** | ***0.36*** |  | ***4.88*** | ***0.40*** | ***4.81*** | ***0.48*** |  |
| 4. Right to freedom from torture, cruel and inhuman treatment and punishment which would detrimentally impact mental wellbeing | ***4.23*** | ***1.10*** | ***4.59*** | ***0.97*** |  | ***4.90*** | ***0.30*** | ***4.78*** | ***0.64*** |  | ***4.95*** | ***0.22*** | ***4.81*** | ***0.48*** |  |
| 5. Right to equal recognition before the law (including equal legal capacity to hold and exercise rights and have decisions legally enforced) | 3.75 | 1.17 | 3.85 | 1.03 |  | 4.21 | 0.98 | 4.04 | 0.76 |  | 4.38 | 1.00 | 4.15 | 0.77 |  |
| 6. Right to effective access to justice, including accommodations to participate in justice and legal proceedings | ***4.18*** | ***1.01*** | 3.96 | 1.06 |  | 4.46 | 0.64 | 4.15 | 0.82 |  | 4.55 | 0.64 | 4.26 | 0.76 |  |
| 7. Right to health, including access to health services/appropriate treatment | ***4.31*** | ***0.92*** | ***4.44*** | ***0.85*** |  | ***4.81*** | ***0.40*** | ***4.52*** | ***0.70*** |  | ***4.89*** | ***0.31*** | ***4.56*** | ***0.70*** | * |
| 8. Right to consent to treatment | 3.93 | 1.27 | 3.63 | 1.18 |  | 4.36 | 1.04 | 3.78 | 1.01 | ** | 4.38 | 1.03 | 4.07 | 1.00 |  |
| 9. Right to challenge potential rights violation before a judicial body or committee | 4.18 | 0.98 | 4.07 | 1.14 |  | 4.36 | 0.74 | 4.30 | 0.87 |  | 4.53 | 0.60 | 4.41 | 0.84 |  |
| 10. The right to work and workplace equality | 3.95 | 1.12 | 3.74 | 0.90 |  | 4.45 | 0.60 | 3.96 | 0.72 | ** | 4.61 | 0.55 | 4.26 | 0.71 | * |
| 11. The right to provision of services and programmes that enable the attainment and maintenance of independence, capability, inclusion and participation in all aspects of life | 3.90 | 1.10 | 3.70 | 1.35 |  | 4.49 | 0.64 | 4.22 | 0.93 |  | 4.70 | 0.52 | 4.41 | 1.01 |  |
| 12. Right to education | 4.15 | 0.93 | 4.15 | 0.99 |  | 4.54 | 0.72 | 4.15 | 0.82 | * | 4.62 | 0.63 | 4.30 | 0.67 | * |
| 13. Right to adequate living standards | 4.00 | 1.15 | 3.93 | 1.17 |  | ***4.66*** | ***0.58*** | 4.33 | 0.73 |  | ***4.72*** | ***0.51*** | 4.48 | 0.70 |  |
| 14. Right to social inclusion and participation in community life (including right to (re)habilitation) | 3.95 | 1.18 | 4.22 | 1.09 |  | 4.51 | 0.60 | ***4.48*** | ***0.80*** |  | 4.68 | 0.62 | ***4.59*** | ***0.57*** |  |
| 15. Right to measures which facilitate independent living | 3.98 | 1.00 | 3.42 | 1.21 |  | 4.41 | 0.64 | 4.04 | 0.82 |  | 4.58 | 0.59 | 4.08 | 0.89 | * |
| 16. Right to personal physical mobility | 3.93 | 1.05 | 3.88 | 0.91 |  | 4.32 | 0.66 | 4.08 | 0.84 |  | 4.55 | 0.55 | 4.19 | 0.69 | * |
| 17. Right to participation in cultural life | 3.92 | 1.01 | 3.88 | 1.24 |  | 3.95 | 0.90 | 4.08 | 1.02 |  | 4.20 | 0.79 | 4.27 | 1.00 |  |
| 18. Right to participation in political life | 4.00 | 1.09 | 3.63 | 1.04 |  | 4.13 | 0.91 | 3.89 | 0.75 |  | 4.40 | 0.74 | 3.93 | 0.83 | * |
| 19. Right to protection and safety in emergency situations | ***4.20*** | ***0.97*** | ***4.37*** | ***0.84*** |  | ***4.67*** | ***0.58*** | ***4.52*** | ***0.80*** |  | ***4.78*** | ***0.48*** | ***4.67*** | ***0.62*** |  |
| 20. Right to freedom of expression, and access to information | 4.15 | 0.87 | ***4.33*** | ***1.00*** |  | 4.34 | 0.67 | 4.33 | 0.88 |  | 4.46 | 0.64 | 4.52 | 0.75 |  |
| 21. Right to consent to participate in research | ***4.30*** | ***0.94*** | ***4.52*** | ***0.80*** |  | 4.34 | 0.81 | 4.37 | 0.84 |  | 4.45 | 0.71 | 4.52 | 0.75 |  |
