# Supplementary material for: Assessing the priority of human rights and mental health: the PHRAME approach
Source: BJPsych Open. 2023 Mar 27;9(2):e56. doi: 10.1192/bjo.2023.41 (PMC10134285; doi:10.1192/bjo.2023.41)
Supplement: Supplementary file 1 [file bjosup.zip › S2056472423000418sup004.pdf]

Assessing the priority of human rights and mental health: the PHRAME approach

Petra C Gronholm, Neeraj S Gill, Grace Carter, Danielle Watson, Hanfried Helmchen, Graham Thornicroft*, Norman Sartorius* (*Joint senior authors)
Corresponding author: Petra Gronholm, email: petra.gronholm@kcl.ac.uk

---

Supplementary Material file 2: Post-hoc analysis results

Table: Key human rights of people with mental illness, as assessed in terms of feasibility, urgency, and overall importance, across the whole sample, arranged per participants' region of work. ***Bold italics*** = top five scores with strongest agreement. *≤0.05; **≤0.01

Key human rights statements	FEASIBILITY					URGENCY					OVERALL IMPORTANCE				
	High income		Other			High income		Other			High income		Other		
	mean	SD	mean	SD	p	mean	SD	mean	SD	p	mean	SD	mean	SD	p
1. Right to equality in all aspects of the law	3.93	1.07	4.15	0.99		4.31	0.86	4.19	0.74		4.46	0.84	4.35	0.69	
2. Right to freedom from discrimination in accessing rights on the grounds of disability	4.13	0.82	4.19	0.88		4.58	0.59	4.37	0.56		4.66	0.53	4.48	0.58	
3. Right to freedom from exploitation, violence, and abuse	4.00	1.13	4.30	1.14		***4.78***	***0.53***	***4.85***	***0.36***		***4.88***	***0.40***	***4.81***	***0.48***	
4. Right to freedom from torture, cruel and inhuman treatment and punishment which would detrimentally impact mental wellbeing	***4.23***	***1.10***	***4.59***	***0.97***		***4.90***	***0.30***	***4.78***	***0.64***		***4.95***	***0.22***	***4.81***	***0.48***	
5. Right to equal recognition before the law (including equal legal capacity to hold and exercise rights and have decisions legally enforced)	3.75	1.17	3.85	1.03		4.21	0.98	4.04	0.76		4.38	1.00	4.15	0.77	
6. Right to effective access to justice, including accommodations to participate in justice and legal proceedings	***4.18***	***1.01***	3.96	1.06		4.46	0.64	4.15	0.82		4.55	0.64	4.26	0.76	
7. Right to health, including access to health services/appropriate treatment	***4.31***	***0.92***	***4.44***	***0.85***		***4.81***	***0.40***	***4.52***	***0.70***		***4.89***	***0.31***	***4.56***	***0.70***	*
8. Right to consent to treatment	3.93	1.27	3.63	1.18		4.36	1.04	3.78	1.01	**	4.38	1.03	4.07	1.00	
9. Right to challenge potential rights violation before a judicial body or committee	4.18	0.98	4.07	1.14		4.36	0.74	4.30	0.87		4.53	0.60	4.41	0.84	
10. The right to work and workplace equality	3.95	1.12	3.74	0.90		4.45	0.60	3.96	0.72	**	4.61	0.55	4.26	0.71	*
11. The right to provision of services and programmes that enable the attainment and maintenance of independence, capability, inclusion and participation in all aspects of life	3.90	1.10	3.70	1.35		4.49	0.64	4.22	0.93		4.70	0.52	4.41	1.01	
12. Right to education	4.15	0.93	4.15	0.99		4.54	0.72	4.15	0.82	*	4.62	0.63	4.30	0.67	*
13. Right to adequate living standards	4.00	1.15	3.93	1.17		***4.66***	***0.58***	4.33	0.73		***4.72***	***0.51***	4.48	0.70	
14. Right to social inclusion and participation in community life (including right to (re)habilitation)	3.95	1.18	4.22	1.09		4.51	0.60	***4.48***	***0.80***		4.68	0.62	***4.59***	***0.57***	
15. Right to measures which facilitate independent living	3.98	1.00	3.42	1.21		4.41	0.64	4.04	0.82		4.58	0.59	4.08	0.89	*
16. Right to personal physical mobility	3.93	1.05	3.88	0.91		4.32	0.66	4.08	0.84		4.55	0.55	4.19	0.69	*
17. Right to participation in cultural life	3.92	1.01	3.88	1.24		3.95	0.90	4.08	1.02		4.20	0.79	4.27	1.00	
18. Right to participation in political life	4.00	1.09	3.63	1.04		4.13	0.91	3.89	0.75		4.40	0.74	3.93	0.83	*
19. Right to protection and safety in emergency situations	***4.20***	***0.97***	***4.37***	***0.84***		***4.67***	***0.58***	***4.52***	***0.80***		***4.78***	***0.48***	***4.67***	***0.62***	
20. Right to freedom of expression, and access to information	4.15	0.87	***4.33***	***1.00***		4.34	0.67	4.33	0.88		4.46	0.64	4.52	0.75	
21. Right to consent to participate in research	***4.30***	***0.94***	***4.52***	***0.80***		4.34	0.81	4.37	0.84		4.45	0.71	4.52	0.75	
